# Treatment of Tonsillitis

**Published:** 1893-09-09

**Authors:** 


### BRISTOL ROYAL INFIRMARY.

### The Treatment of Tonsillitis.

Before passing on to the treatment adopted in various forms of acute and chronic inflammatory affections of the tonsil, a few remarks on the structure and functions of the tonsils may be interesting.

Now, we are not altogether clear as to the exact nature of the functions of the tonsil, but an examination of its structure shows that a tonsil is simply an aggregation of lymphoid tissue, with a reduplicated or involuted surface, forming crypts which were formerly supposed to be the orifices of secretory glands, a view no longer tenable. Similar aggregations of adenoid tissue are formed at the extreme base of the tongue on its dorsal surface, and on the roof and posterior wall of the post-nasal space.

Such adenoid tissue is exactly analogous, and we believe almost identical in function with the aggregations of this tissue in the intestines, and known as Peyer's patches and solitary glands. We have good reason to believe that various toxic matters, and especially micro-organisms, which gain access to the tonsillar crypts, and Peyer's patches, are seized, destroyed and disintegrated, and thus prevented in health from exercising a deleterious action on the organism.

It is interesting that diphtheria, catarrhal and follicular tonsillitis, and peritonsillitis, scarlet fever, are especially diseases of children, in whom all the tonsils are functionally most active, while in adults, at a time of life when the tonsils have generally atrophied, such diseases are less common. That there is a connection between acute tonsillitis and rheumatism there can be no manner of doubt, but our experience accords with the views of McBride, when he says, "As to the etiology of tonsillitis I strongly suspect that in future years we shall come to recognise the fact that the same disease may be produced by very different factors, and further, that what appears to be the same disease may have quite a different life history. I have little doubt that there is a very distinct connection between the rheumatic diathesis and certain forms of tonsillitis. I have equally little doubt that there is a very intimate connection between another form of tonsilitis (not at present distinguishable from the rheumatic form) and diphtheria. It is also probable that still another variety of the same complaint is an infectious disease *sui generis*, as held by Fränkel and Seifert. Finally we have seen that a form of tonsillitis is very commonly associated with, and due to, septic influences. . . . If, when a case of tonsillitis occurs, there happens, as is not unusual in our cities, to be a prevalence of scarlatina, the etiology of the affection may be still further complicated. No doubt inflammation of the tonsils is more liable to occur during cold and wet weather; this applies both to the suppurative and non-suppurative forms. Whether any other diathesis besides the rheumatic plays an important part in the etiology of tonsillitis is doubtful. The disease occurs more commonly in young adults, but is seen both in children and in elderly people. The attack is often ascribed directly to a chill, while chronic enlargement of the tonsils seems to act as a predisposing cause. To recapitulate, we may have: (1) A rheumatic tonsillitis; (2) a diphtheritic tonsillitis; (3) a contagious tonsillitis; (4) a septic tonsillitis; (5) a suppurative peri-tonsillitis, a tonsillitis sometimes secondary to one of the preceding forms and sometimes primary."

As regards the treatment of acute tonsillitis adopted in Dr. Watson Williams' clinic, we shall here only refer to the rheumatic forms of tonsillitis.

Excluding diphtheria, scarlatina, and other exanthemata, and septic affections, all acute inflammations of the tonsils are regarded as rheumatic and treated accordingly by a mixture of salicylate of soda, grs. xx. to the ounce of water, to which Tr. nucis. vom. ♏i. ii. are added to overcome the nauseous sweetness of the soda salt. If the patient is an out-patient this is given every four hours, but in the house it is given every two hours for a few doses, and then less frequently. It is very rarely that no improvement follows in the course of five or six hours, and generally the pain is greatly relieved. The bowels are freely moved by a saline aperient, and any tendency to constipation treated by suitable remedies.

The pain on swallowing is practically obviated if the patient firmly compresses the ears just in front of the external orifice of the meatus during deglutition.

Salol, salicin, and salicylic acid are never prescribed now in this department in the treatment of rheumatic tonsilitis, their action being found to be inferior to the salicylate of soda, and less certain.

Tincture of aconite is sometimes given where the temperature is considerably raised in doses of one drop every half-hour for a few hours, and then every two hours. This remedy, although so well known for its action in reducing temperature and modifying the cause of acute catarrhal tonsilitis, is now almost completely given up in favour of salicylate of soda.

If suppuration occurs in the tonsil, the salicylate of soda may be discontinued with advantage, and iron or iron and quinine given instead.

As the acute febrile stage passes off the patient will require tonics and plenty of nourishing food, as an attack of tonsillitis always reduces the sufferer considerably.

*Local Treatment.*—The pain in the throat will be greatly relieved by hot fomentations with tincture of belladonna or laudanum, and these may be preceded by a linseed poultice with mustard applied to the under surface of the chin, extending round to the region of the tonsils on either side.

As regards applications to the tonsils, a lozenge containing one-eighth of a grain of cocaine and a few grains of chlorate of potash will be grateful, providing swallowing the saliva is not too painful. When the pain is great a gargle of chlorate of potash and bicarbonate of soda is useful, and causes less discomfort than the continual swallowing necessitated in sucking a lozenge.

Spraying the throat three or four times a day with sanitas, one part in four of water, with thirty minims of glycerine to the ounce, is found to exercise a beneficial influence on the inflammation and exudation of the tonsil. If the infiltration and swelling be very considerable the tonsil should be freely punctured with incisions a quarter of an inch deep, and bleeding encouraged. The relief to the congested tonsil is often very marked, and suppuration may sometimes be prevented.

If suppuration has actually occurred, the pus should be evacuated by a deep incision made from without inwards with a curved pointed bistoury, so as to avoid risk of injuring the carotid artery, which lies well to the outer side of the tonsil.

The tonsils should never be removed while acutely inflamed, unless in the very rare cases where the enlargement and swelling is so great as to greatly embarrass respiration.

On the one hand, patients are often unduly alarmed by the symptoms of acute tonsillitis; the practitioner, on the other hand, is sometimes liable to under-estimate the gravity of the remoter possibilities of the affection. A case was recently recorded in which tonsillitis ended in death by suffocation from rupture of a tonsillar abscess with the escape of pus into the larynx, a condition confirmed by post-mortem examination.

As the condition passes into the subacute stage the tonsils may be painted once daily with the ordinary tincture of perchloride of iron, but local treatment is then much less important than attention to the general health, and overcoming the tendency to atonic dyspepsia that is so often left. But repeated attacks are sure to give rise to a chronic enlargement of the tonsils, which are the seat of continually recurrent slight inflammation, and materially interfere with the patient's wellbeing. We will refer to the treatment of chronic inflammation of the tonsils in a future article.

